# Increased Voluntary Alcohol Consumption in Mice Lacking GABA_B(1)_ Is Associated With Functional Changes in Hippocampal GABA_A_ Receptors

**DOI:** 10.3389/fnbeh.2022.893835

**Published:** 2022-06-09

**Authors:** Gabriele Floris, Gino Paolo Asuni, Giuseppe Talani, Francesca Biggio, Maria Giuseppina Pisu, Mary Tresa Zanda, Liliana Contu, Elisabetta Maciocco, Mariangela Serra, Paolo Follesa

**Affiliations:** ^1^Department of Life and Environment Sciences, Section of Neuroscience and Anthropology, University of Cagliari, Cagliari, Italy; ^2^Institute of Neuroscience-Cagliari, National Research Council, Cagliari, Italy; ^3^Center for Substance Abuse Research, Lewis Katz School of Medicine, Temple University, Philadelphia, PA, United States

**Keywords:** GABA_B_ receptor, extrasynaptic GABA_A_Rs, functional crosstalk, alcoholism, neuroactive steroids, steroidogenesis, allopregnanolone, hippocampus

## Abstract

Gamma-aminobutyric acid type B receptor (GABA_B_R) has been extensively involved in alcohol use disorders; however, the mechanisms by which this receptor modulates alcohol drinking behavior remain murky. In this study, we investigate alcohol consumption and preference in mice lacking functional GABA_B_R using the 2-bottle choice paradigm. We found that GABA_B(1)_, knockout (KO), and heterozygous (HZ) mice drank higher amounts of an alcoholic solution, preferred alcohol to water, and reached higher blood alcohol concentrations (BACs) compared to wild-type (WT) littermates. The GABA_B_R agonist GHB significantly reduced alcohol consumption in the GABA_B(1)_ HZ and WT but not in the KO mice. Next, because of a functional crosstalk between GABA_B_R and δ-containing GABA_A_ receptor (δ-GABA_*A*_R), we profiled δ subunit mRNA expression levels in brain regions in which the crosstalk was characterized. We found a loss of the alcohol-sensitive GABA_A_R δ subunit in the hippocampus of the GABA_B(1)_ KO alcohol-naïve mice that was associated with increased ɣ^2^ subunit abundance. Electrophysiological recordings revealed that these molecular changes were associated with increased phasic inhibition, suggesting a potential gain of synaptic GABA_A_R responsiveness to alcohol that has been previously described in an animal model of excessive alcohol drinking. Interestingly, voluntary alcohol consumption did not revert the dramatic loss of hippocampal δ-GABA_A_R occurring in the GABA_B(1)_ KO mice but rather exacerbated this condition. Finally, we profiled hippocampal neuroactive steroids levels following acute alcohols administration in the GABA_B(1)_ KO and WT mice because of previous involvement of GABA_B_R in the regulation of cerebral levels of these compounds. We found that systemic administration of alcohol (1.5 g/kg) did not produce alcohol-induced neurosteroid response in the GABA_B(1)_ KO mice but elicited an expected increase in the hippocampal level of progesterone and 3α,5α-THP in the WT controls. In conclusion, we show that genetic ablation of the GABA_B(1)_ subunit results in increased alcohol consumption and preference that were associated with functional changes in hippocampal GABA_A_R, suggesting a potential mechanism by which preference for alcohol consumption is maintained in the GABA_B(1)_ KO mice. In addition, we documented that GABA_B(1)_ deficiency results in lack of alcohol-induced neurosteroids, and we discussed the potential implications of this finding in the context of alcohol drinking and dependence.

## Introduction

Alcohol use disorder (AUD) is one of the largest public health problems worldwide, with devastating socioeconomic repercussions. In 2016, 2.8 million deaths were attributable to alcohol use and misuse, accounting for nearly 5% of all global deaths (GBD 2016 Alcohol and Drug Use Collaborators, 2018). To curb this epidemic, identification of effective strategies is needed to achieve AUD prevention and therapy. A considerable number of preclinical and clinical evidence has indicated the ɣ-aminobutyric acid (GABA) inhibitory system as one of the most promising targets for the effective treatment of AUD (Addolorato et al., 2012). The GABAergic system is the primary inhibitory neurotransmitter in the brain that fine-tunes neuronal excitability by preventing hyperexcitation (Olsen and Sieghart, 2008). The GABA-mediated inhibitory action is produced by two different classes of receptors that diverge in terms of structural, physiological, and pharmacological properties. GABA type A receptors (GABA_A_Rs) are a family of chloride ion channels that mediate fast inhibitory signals by hyperpolarization of the postsynaptic membrane, whereas GABA type B receptors (GABA_B_Rs) are G protein-coupled metabotropic receptors that produce slow and persistent inhibitory signals (Bettler et al., 2004). The ionotropic GABA_A_R has been largely involved in the acute and chronic effects of alcohol in the brain including pharmacological tolerance, dependence, and withdrawal symptoms (Enoch, 2008; Kumar et al., 2009; Olsen and Liang, 2017). Now, there is also considerable evidence supporting the involvement of metabotropic GABA_B_R in alcohol-drinking behaviors and dependence.

Pharmacological studies have documented that activation of GABA_B_R by the prototypical receptor agonist baclofen and/or ɣ-hydroxybutyric acid (GHB), resulted in reduction of alcohol-drinking behaviors in animal models and in human patients with AUD; therefore, both drugs have been consistently proposed as antialcohol therapy (Biggio et al., 1992; Caputo et al., 2009; Addolorato et al., 2011; Agabio et al., 2018; Colombo and Gessa, 2018). A genetic study has found that a single nucleotide polymorphism in the GABA_B(1)_ promoter is predictive of AUD in human populations (Enoch et al., 2016). Aberrant splicing of GABA_B(1)_ has been found in post-mortem human brain samples from alcoholics (Lee et al., 2014), suggesting that both genetic and epigenetic regulations of GABA_B(1)_ expression may increase AUD risk. A recent RNA-seq study investigating GABAergic gene expression in post-mortem patients with AUD and cocaine addicts have revealed a robust downregulation of hippocampal GABA_B(1)_ (Enoch et al., 2012). In the same study, diminished expression of GABA_B(1)_ was also found in the hippocampus of alcohol-naïve and alcohol-preferring rats, suggesting that diminished expression and function of hippocampal GABA_B_R may be a predisposing factor for drug and alcohol dependence irrespective of species (Enoch et al., 2012). Nonetheless, the mechanisms by which GABA_B_R modulates alcohol drinking behaviors and favor alcohol dependence remain unclear.

A potential mechanism by which GABA may regulate alcohol drinking has emerged from recent studies showing the role of GABA_B_R in modulating tonic currents that are mediated by extrasynaptic δ-containing GABA_A_R (δ-GABA_A_R) (Stell et al., 2003; Glykys et al., 2008; Carver and Reddy, 2016). It has been found that pharmacological activation of GABA_B_R increases tonic currents mediated byδ-GABA_A_ in thalamic relay neurons, cerebellar granule cells, and hippocampal dentate gyrus granule cells (DGGCs) (Connelly et al., 2013; Tao et al., 2013; Khatri et al., 2019). Extrasynaptic δ-GABA_A_Rs have been extensively associated with the pharmacological effect of alcohol on the brain (Mihalek et al., 2001; Sundstrom-Poromaa et al., 2002; Wallner et al., 2003; Wei et al., 2004), and significant changes in alcohol drinking behaviors have been reported by pharmacological and genetic manipulation of the GABA_A_R δ subunit (Nie et al., 2011; Ramaker et al., 2011; Fritz and Boehm, 2014; Melón et al., 2019). These findings raise the possibility that some effects of GABA_B_R agonists on alcohol drinking and related behaviors might be mediated, at least in part, by modulating δ-GABA_A_R activity.

Based on this background, we hypothesized that genetic ablation of the fundamental GABA_B(1)_ subunit of GABA_B_ R may increase alcohol drinking behaviors. Thus, we investigated alcohol drinking behavior in mice lacking functional GABA_B_R (Schuler et al., 2001). Moreover, based on recent findings supporting a functional interaction between GABA_B_ and δ-GABA_A_ receptors, we hypothesized that lack of GABA_B_R may produce functional changes in “alcohol-sensitive” receptors. To test this hypothesis, we studied the expression and function of extrasynaptic δ-GABA_A_ Rs in different brain areas of alcohol-naïve and, subsequently, alcohol-exposed mice. Finally, because of involvement of GABA_B_R in regulating brain levels of neuroactive steroids (Barbaccia et al., 2002), an important class of endogenous modulators of extrasynaptic δ-GABA_A_R with an established role in mediating several behavioral effects produced by alcohol (Morrow et al., 2006; Reddy, 2010; Porcu and Morrow, 2014), we profiled hippocampal neuroactive steroids levels following acute alcohol administration in GABA_B(1)_ knockout (KO) and wild-type (WT) mice.

## Materials and Methods

### Subjects

Balb/c GABA_B(1)_ KO mice were generously provided by Dr. Martin Gassmann of University of Basel, Switzerland, and have been previously characterized by Schuler et al. (2001). Over the years, the mouse colony was back-crossed every 20 generations (F20) with a Friend Virus B (FVB) NIH Jackson mouse strain to obviate genetic drift (Fattuoni et al., 2020). All the subjects were kept in our animal facility with an artificial 12:12 light/dark cycle and constant temperature (23°C) and humidity (65%); food and water were provided *ad libitum*. On postnatal day 21, litters were weaned and genotyped by polymerase chain reaction (PCR) using specific primers and thermocycler conditions as follow: primer 1 targeting exon 10 (forward) 5′AGCTGACCAGACCTTGGTCAT ′3; primer 2 targeting exon 11 (reverse) 5′ AACTGGCTTCTCCCTATGTGG ′3; primer 3 NeoStart (reverse) 5′ATGGGATCGGCCATTGAACAA ′3; initial incubation at 95°C for 5 min, 35 cycles of (denaturation at 95°C for 1 min, annealing at 60°C for 1 min, and extension at 72°C for 1 min), final extension at 72°C for 5 min. Only male subjects were used in all the experiments. After weaning, experimental subjects were group-housed in standard propylene mouse cages (from 3 to 6 subjects/cage) and kept in a dedicated room away from the mating area. All the mice were euthanized (in the dark if needed). This study was performed in accordance with the recommendations of the “Guidelines for Care and Use of Experimental Animals” issued by the Italian Ministry of Health (D.L. 26/2014) and the European Union (2010/63/UE), and the “Guide for Care and Use of Laboratory Animals” adopted by NIH, United States (eighth edition, 2011). The University of Cagliari “Committee of Animal Use and Care” read and approved the experimental protocol.

### Drugs

Absolute ethanol (alcohol) was purchased from Carlo Erba Reagents SRL (Milano, Italy). Alcohol solutions were prepared with filtered water; alcohol was dissolved in a sterile saline solution (sodium chloride 0.9%) for intraperitoneal (IP) injection. GHB sodium salt was purchased from Sigma-Aldrich (St. Louis, MO, United States) and dissolved in the same sterile saline solution for IP injection. The dose of GHB in all the experiments was 100 mg/kg, while the dose of alcohol for IP injection was 1.5 mg/kg. GHB was injected at a volume of 10 ml/kg 15 min prior to beginning the test; alcohol was injected at a volume of 10 ml/kg 60 min before euthanasia and neuroactive steroids measurement. Control mice in all the experiments received an equivalent volume of saline solution.

### Limited Access 2-Bottle Choice Drinking Paradigm

**FIGURE 1:**
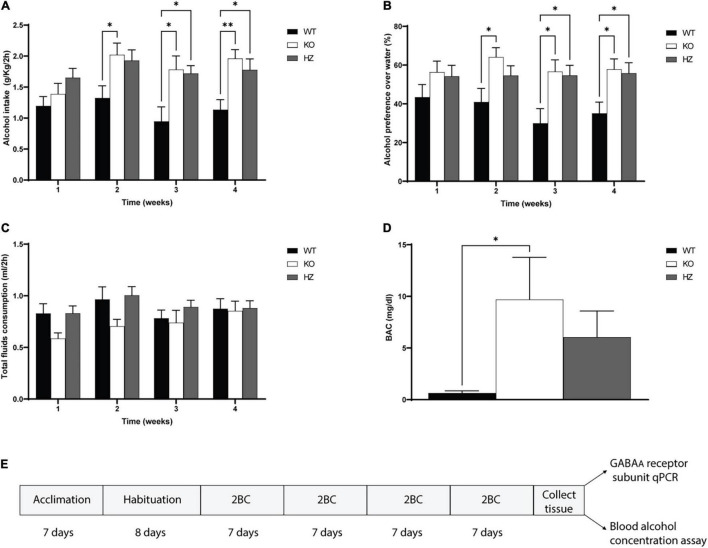
Limited access 2-bottle choice (2BC) drinking paradigm to study alcohol intake, preference, total fluid consumption, and blood alcohol concentration (BAC). **(A)** Total alcohol intake in GABA_B(1)_ WT, KO, and HZ mice during the total period (4 weeks) of 2BC exposition (WT, *n* = 13; KO, *n* = 9; HZ, *n* = 15). **(B)** Alcohol preference to water during the 4 weeks of 2BC; (WT, *n* = 13; KO, *n* = 9; HZ, *n* = 15). **(C)** Total fluid consumption recorded in GABA_B(1)_ WT, KO, and HZ during the 2BC drinking paradigm; (WT, *n* = 13; KO, *n* = 9; HZ, *n* = 15). **(D)** BAC in GABA_B(1)_ WT, KO, and HZ mice was determined at the end of the last drinking session (WT, *n* = 13; KO, *n* = 8; HZ, *n* = 14). *Post hoc* test (^**^p < 0.01; *p < 0.05). **(E)** Schematic representation of experimental timeline. All data are expressed as mean ± SEM.

Thirteen weeks old mice were placed in a dedicated room with 12:12 inverted light/dark cycle and allowed to adapt to the new light/dark cycle for 1 week before the beginning of testing. We used a modified sucrose-fading procedure as we have previously described (Sanna et al., 2011; Follesa et al., 2015). Briefly, the mice were provided with daily access to an alcohol solution for 2 h, beginning 30 min prior to the dark cycle start point. Bottles containing the alcohol solution and filtered water as alternative fluid were offered for a total of 36 days. During the first 8 days of the habituation period, the mice received the following alcohol/sucrose (v/v; w/v) solutions: days 1–2, 10–5%; days 3–4, 12.5–4%; days 5–6, 15–2%; day, 7–8, 15–0%. For the rest of the experiment, the mice were maintained on 15% (v/v) alcoholic solution sucrose-free. Solutions were offered at room temperature and prepared fresh twice a week, and bottle positions were switched daily to avoid side preference. The mice were individually housed during the *2 BC* and group-housed at the end of a drinking session. Food and water were provided *ad libitum* throughout the entire paradigm, and mouse body weight was recorded twice a week. To evaluate the amount of alcohol and water consumed, bottles were weighted before and after a drinking session. An additional control group for molecular analysis was kept under the same experimental conditions, but alcohol-containing bottles were replaced with water. A schematic representation of experimental timeline is depicted in Figure 1E.

### Limited Access 2-Bottle Choice Drinking Paradigm and GHB Treatment

We performed an independent experiment using the same 2 h limited access 2BC drinking paradigm described above. Following the 8 days of habituation period, we divided the subjects for each genotype in two experimental groups (GHB and saline). Then, we treated the mice daily with GHB or saline 15 min before the beginning of the drinking session. Drinking behaviors were evaluated for 10 consecutive days.

### Spontaneous Locomotor Activity

Spontaneous locomotors activity was performed as we have previously described in Sanna et al. (2011). Briefly, the mice were exposed to the experimental room for 2 h prior the beginning of the experiment and then received an IP injection of GHB or saline 15 min before beginning the test. Spontaneous locomotor activity was assessed by placing the subjects in the center of the 25 cm × 25 cm square arena of an infrared Actimeter (Harvard Apparatus, MA, United States). The subjects were left undisturbed to freely move for a total time of 60 min. After each session, the arena was thoroughly cleaned with 70% alcohol.

### Blood Alcohol Concentration Assay

Immediately after decapitation, blood samples (140 μl) were collected from the trunk with two heparinized capillary tubes, transferred to 1.5-ml test tubes containing 10 μl heparin, and mixed; 100 μl aliquots of blood was then subjected to BAC analysis by static headspace capillary gas chromatography/mass spectrometry following the same protocol that we previously described in Follesa et al. (2015). BAC values were expressed in mg/dl units.

### Measurement of GABA_A_R Subunit mRNA Level by qRT-PCR

Brain areas were rapidly removed following the decapitation and placed in 1.5-ml RNase-free plastic tubes. Liquid nitrogen was used to snap-freeze the dissected tissues; frozen tissues were then stored at –80°C until the day of analysis. Total RNA isolation was performed using TRI Reagent^®^ (Sigma-Aldrich, St. Louis, MO, United States) following the manufacturer’s instructions. The RNA concentration of each sample was determined by measuring the absorbance at 260 nm. From each sample, 1 μg of RNA was employed for single-strand cDNA synthesis using an i*Script*™ cDNA synthesis kit (Bio-Rad, Hercules, CA, United States) according to the manufacturer’s instructions. Diluted cDNA (1:50) was used as a template for quantitative RT-PCR to determine GABA_A_R subunit mRNA levels using the same experimental protocol and thermocycler conditions we previously described in Follesa et al. (2015). The primer assay “QuantiTect” from Qiagen (Hilden, Germany) was used for measuring the following GABA_A_R subunits and housekeeping genes: GABA_A_R α4 (product number 249900, NM_010251, final conc. 1X), GABA_A_R δ (product number 249900, NM_008072, final conc. 1X), GABA_A_R ɣ^2^ (product number 249900, NM_008073; NM_177408, final conc. 1X), beta-actin (product number 249900, NM_007393, final conc. 1X), and GAPDH (product number 249900, NM_008084, final conc. 1X).

### Electrophysiology

Brain slice preparation and whole-cell patch-clamp recordings were performed as previously described (Sanna et al., 2011). In brief, recordings were made from neurons held at –65 mV using borosilicate glass pipettes (resistance, 2.5–4.5 MΩ) that were pulled with a Flaming/Brown micropipette puller (Sutter Instruments, Novato, CA, United States) and containing the following (in mM): 150 CsCl, 10 HEPES, 5 lidocaine N-ethyl bromide, 2 MgCl2, 3 Mg-ATP, 0.3 Na-GTP, and 10 BAPTA-4K, pH 7.25–7.30 (osmolarity∼298 mOsm). GABA-evoked Cl- currents were recorded by adding kynurenic acid (3 mM) to the extracellular aCSF recording solution. Voltage-clamp was performed with an Axopatch 200-B amplifier (Axon Instruments, Foster City, CA, United States). Recordings were discarded if the access resistance was over 25 MΩ or changed by more than 20%. Data were filtered at 2 kHz and digitized at 5 kHz. Spontaneous inhibitory postsynaptic current (sIPSC) amplitude, frequency, and decay time were acquired using the pClamp 9.2 software (Molecular Devices, CA, United States). Tonic currents were recorded after 6 min of GABA_A_R δ partial agonist 4,5,6,7-tetrahydroisoxazolo[5,4-c]pyridin-3-ol (THIP, Gaboxadol) 3 (μM) application. After THIP perfusion, bicuculline (20 μM) was further added for blocking both phasic and tonic currents. To evaluate the tonic components of GABAergic inhibition, shift in holding current (pA), and change in noise variance (% vs. control) caused by drug perfusion were evaluated. All the reagents and drugs used were purchased from Sigma-Aldrich (St. Louis, MO, United States).

### Extraction and Assay of Neuroactive Steroids

Brains were rapidly removed from the skull following the decapitation, and hippocampi were rapidly dissected and frozen in liquid nitrogen and then stored at −80°C until steroid extraction. Progesterone, 3α-hydroxy-5α-pregnan-20-one (3α,5α-TH-PROG; allopregnanolone, AP), and 3α,5α tetrahydrodeoxycorticosterone (3α,5α-THDOC; THDOC) were extracted and purified as we have previously described (Purdy et al., 1990; Serra et al., 2000). Briefly, neuroactive steroids were extracted with ethyl acetate (three times), and resulting organic phases were dried using a vacuum. The resulting residue was combined in 5 ml of n-hexane and applied to a SepPak silica cartridge (Waters), and then eluted using n-hexane and 2-propanol (7:3 vol/vol). Steroid separation and purification were performed by HPLC on a 5-mm Lichrosorb-diol column (250 3 4 mm; Phenomenex) using a discontinuous gradient of 2-propanol (0–30%) in n-hexane. Progesterone, that coelutes with cholesterol, required an additional purification that was executed by washing twice with 400 ml of water and 200 ml of dimethyl sulfoxide the resulting HPLC, previously dried, fractions. Lastly, progesterone was extracted twice with 1.5-ml volumes of n-hexane in the aqueous phase. Neuroactive steroids from bilateral hippocampi were quantified by radioimmunoassay (RIA) as previously described (Barbaccia et al., 1996). Specific antibodies against progesterone were purchased from ICN (Costa Mesa, CA, United States) and antibodies to THDOC and AP were generated, respectively, from rabbits and sheep and have been previously characterized (Purdy et al., 1990).

### Statistical Analysis

All data are presented as mean ± SEM and were analyzed by appropriate one- or two-way analysis of variance (ANOVA) or Student’s *t*-test as described in the Section “Results.” The GraphPad Prism 9.0 software was used for all the statistical analyses. Significant main effects and interactions (*p* < 0.05) in the ANOVA were followed by *post hoc* test analysis. For all the behavioral and molecular experiments, Tukey’s multiple comparisons *post hoc* test was conducted with the exception of the GHB and neurosteroid experiments that were analyzed by Šídák′s multiple comparisons test.

## Results

### Increased Alcohol Consumption, Preference, and BAC in GABA_B(1)_ KO Mice

Thanks to previous preclinical and clinical studies supporting the view that diminished GABA_B_R transmission may increase alcohol drinking behaviors and dependence, we investigated for the first time alcohol drinking behaviors in GABA_B(1)_ KO mice. To this end, we monitored daily alcohol intake in GABA_B(1)_ KO, HZ, and WT male mice exposed to the 2-bottle choice drinking paradigm for a total period of 4 weeks. For each subject, daily alcohol intake values (expressed in g/kg/2h) were averaged for each week and grouped by genotype. We found that the GABA_B(1)_ KO and HZ mice drank more alcohol than the WT mice. The two-way repeated measure ANOVA revealed a significant main effect of genotype [*F*_(2,34)_ = 6.872, *p* = 0.0031] and time [*F*_(2.733,92.93)_ = 4.314, *p* = 0.0085] but not genotype × time interaction [*F*_(6,102)_ = 1.341, *p* = 0.2458]. Tukey’s multiple comparisons test showed significantly higher alcohol intake in the GABA_B(1)_ KO mice starting on week 2 (WT vs. KO *p* = 0.0473) and maintained over time (WT vs. KO *p* = 0.0433; *p* = 0.0031, weeks 3 and 4). Similarly, the HZ mice showed increased alcohol drinking starting on week 3 (WT vs. HZ *p* = 0.0231) that was also maintained on week 4 (WT vs. HZ *p* = 0.032) (Figure 1A). Lack and/or reduction of GABA_B_R function resulted in overall increase in alcohol preference to water. Alcohol preference scores were calculated as the ratio of alcohol intake to total fluid intake [alcohol intake/(alcohol intake + water intake)]. The two-way repeated measure ANOVA with genotype as the between-subject factor and time as the within-subject factor showed a significant main effect of genotype [*F*_(2,34)_ = 5.303, *p* = 0.0099] but not time [*F*_(1.935,65.78)_ = 1.176, *p* = 0.3138] on alcohol preference and no interaction between these factors [*F*_(6,102)_ = 0.9134, *p* = 0.4885]. Tukey’s multiple comparisons test revealed that the GABA_B(1)_ KO mice showed increased alcohol preference compared to WT starting on week 2 (*p* = 0.0338) that was maintained over the weeks (*P* = 0.0315, *P* = 0.0259; weeks 3 and 4, respectively). The HZ mice showed increased alcohol preference compared to WT starting on week 3 (*p* = 0.0343) that was maintained during week 4 (*p* = 0.0365) (Figure 1B). We annotated the total fluid consumption, calculated as the sum of alcohol intake + water intake (ml). The two-way repeated measure ANOVA revealed a main effect of time [*F*_(2.154,73.24)_ = 3.097, *p* = 0.0475] but not genotype [*F*_(2,34)_ = 1.647, *p* = 0.2077] on total fluid consumption; moreover, no interaction was found between genotype and time [*F*_(6,102)_ = 1.194 *p* = 0.3155] (Figure 1C). Finally, we evaluated whether the increased voluntary alcohol consumption in the GABA_B1_ KO and HZ mice resulted in increased BAC. We profiled BAC right after the end of the last drinking session and found that the GABA_B1_ KO mice had higher BAC levels than the WT mice [one-way ANOVA; *F*_(2,31)_ = 3.704, *p* = 0.0361]. Tukey’s multiple comparisons test revealed a statistical significance between KO and WT (*p* = 0.0335) but not between HZ and WT. The BAC average in the KO and HZ mice was 9.41 and 6.04 mg/dl, respectively, whereas the BEC average in the WT mice was only 0.63 mg/dl (Figure 1D).

### ɣ-Hydroxybutyric Acid Decreases Voluntary Alcohol Consumption in GABA_B(1)_ HZ and WT but Not in KO Mice

**FIGURE 2:**
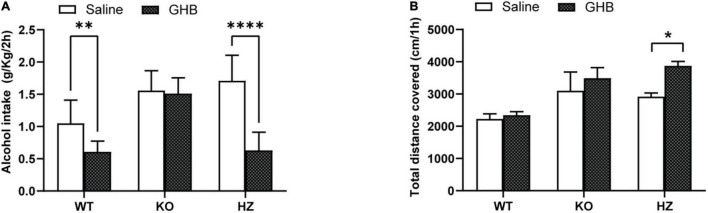
Limited access 2-bottle choice (2BC) drinking paradigm to study the effect of GHB treatment on alcohol consumption in GABA_B(1)_ WT, KO, and HZ mice. **(A)** Alcohol intake in GABA_B(1)_ WT, KO, and HZ mice during the total period of 2BC exposition and GHB or saline treatment (*n* = 5/group). **(B)** Spontaneous locomotor activity was recorded in GABA_B(1)_ WT, KO, and HZ mice; total distance traveled is expressed in cm/1h (WT saline, *n* = 12; KO saline, *n* = 9; HZ saline, *n* = 13; WT GHB, *n* = 11; KO GHB, *n* = 10; HZ GHB, *n* = 14). All mice in panels **(A,B)** received GHB (100 mg/kg) or an equivalent volume of saline 15 min prior to the test. All data are expressed as mean ± SEM. *Post hoc* test (^****^p < 0.0001; ^**^p < 0.01; *p < 0.05).

We evaluated the capability of the GABA_B_R agonist GHB, a drug that has shown anti-alcohol effects on rodents and humans (Biggio et al., 1992; Colombo et al., 2012), to suppress alcohol drinking in our models. To achieve this goal, we treated the GABA_B(1)_ KO, HZ, and WT mice every day with GHB or saline solutions 15 min before a drinking session. The two-way ANOVA showed a main effect of genotype [*F*_(2,54)_ = 27.22, *p* < 0.0001] and treatment [*F*_(1,54)_ = 44.94, *p* < 0.0001] as well as interaction between these two factors [*F*_(2,54)_ = 14.93, *p* < 0.0001]. Multiple comparisons revealed a significant reduction in the voluntary alcohol consumption of HZ and WT mice treated with GHB compared to controls treated with saline (*p* < 0.0001 and *p* = 0.0056, respectively) (Figure 2A). Conversely, GHB treatment in GABA_B(1)_ KO did not produce any effect on alcohol consumption (Figure 2A). To rule out that suppression of alcohol-drinking behaviors after GHB treatment was due to secondary effects (i.e., sedation), we measured the spontaneous locomotor activity in independents groups of mice treated with the same dose of GHB. We found that GHB did not reduce the total distance traveled in all the mice groups. On the contrary, Šídák’s multiple comparisons test revealed an increase in the total distance traveled in the GHB-treated GABA_B(1)_ HZ mice (*p* = 0.0490) (Figure 2B). The two-way ANOVA revealed a main effect of genotype [*F*_(2,63)_ = 12.44, *p* < 0.0001] and treatment [*F*_(1,63)_ = 5.675, *p* = 0.0202], but there was no interaction between these two factors.

### Lack of GABA_B(1)_ Produces Molecular Changes in GABA_A_R Subunit Gene Expression in the Hippocampus

**FIGURE 3:**
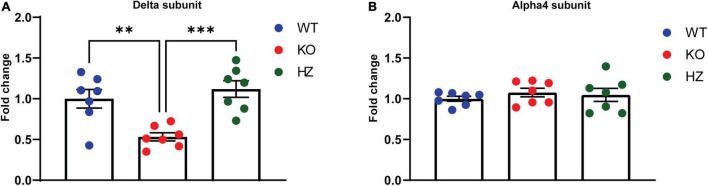
GABA_A_ receptor s measurement in different brain areas revealed a homeostatic change in the hippocampus of GABA_B(1)_ KO. mRNA level of the delta **(A)** and alpha4 **(B)** subunits in the hippocampus of GABA_B(1)_ WT, KO, and HZ mice. *Post hoc* test (^***^p < 0.001; ^**^p < 0.01) (*n* = 7/group). All data are expressed as mean ± SEM.

Recent studies have shown the capability of GABA_B_Rs to directly modulate extrasynaptic δ-GABA_A_R, a subtype of GABA_A_R with an established role in alcohol drinking and dependence. In this study, we sought to expand the knowledge of these interesting findings looking more closely at the molecular level. To this end, we measured the mRNA abundance of the GABA_A_R δ subunit in brain areas where the crosstalk between GABA_B_R and δ-GABA_A_R has been characterized (Connelly et al., 2013; Tao et al., 2013; Khatri et al., 2019). We found a significant decrease in δ subunit mRNA in the hippocampus of the GABA_B(1)_ KO mice compared with both the WT (*p* = 0.0058) and HZ mice (*p* = 0.0008) by one-way ANOVA [*F*_(2,18)_ = 11.27, *p* = 0.0007] followed by Tukey’s multiple comparisons test (Figure 3A). However, we did not find any significant difference in δ subunit mRNA abundance in the cerebellum and thalamus (data not presented). We also extended the analysis to the α4 subunit that forms a specific partnership with δ in this brain region (Wei et al., 2004). We did not observe any changes in gene expression associated with the α4 subunit in all the three genotypes as revealed by the one-way ANOVA [*F*_(2,19)_ = 0.5461, *p* = 0.588] (Figure 3B).

### Lack of GABA_B(1)_ Produces Changes in Phasic but Not Tonic GABAergic Currents in Dentate Gyrus Granule Cells

**FIGURE 4:**
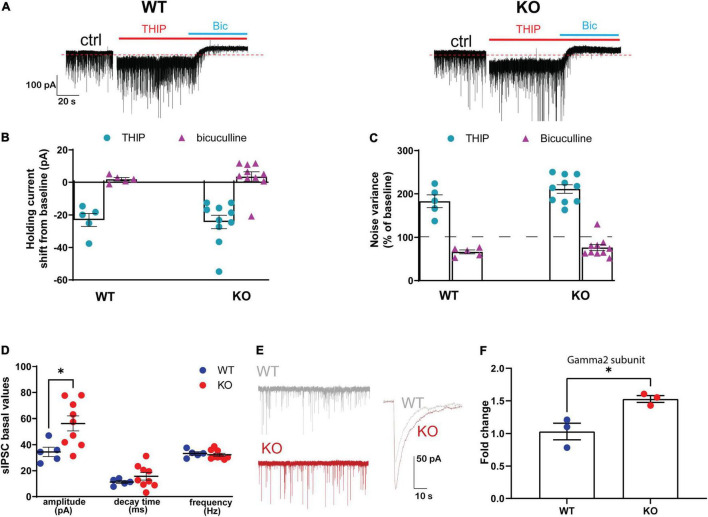
Effect of genetic ablation of GABA_B(1)_ on GABAergic tonic and phasic currents in granule cells of the dentate gyrus. **(A)** Representative traces of whole-cell mode recording of GABAergic tonic currents from GABA_B(1)_ WT (left) and KO (right) mice. After 3 min of baseline, the application of 3 μM THIP increased tonic currents that were blocked by the application of 20 μM bicuculline (Bic). Calibration, 100 pA, 20 s. Panel **(B)** shows the negative shift in the holding current following THIP application (WT, *n* = 5; KO, *n* = 10) and panel **(C)** shows the increase in noise variance compared to the baseline following THIP application (WT, *n* = 5; KO, *n* = 10). All GABAergic currents in panels **(B,C)** were blocked by bicuculline. **(D)** Bar graphs represent the analysis of GABAergic spontaneous IPSCs (sIPSCs), summarizing the changes in sIPSC amplitude expressed in picoampere (pA); decay time is expressed in milliseconds (ms), frequency in hertz (Hz) (WT, *n* = 5; KO, *n* = 9). *Post hoc* test (*p < 0.05). **(E)** Representative trace of sIPSC amplitude highlighting the difference between GABA_B(1)_ WT and KO mice. Calibration 50 pA, 10 s. **(F)** mRNA level of the Gamma2 subunit in the hippocampus of alcohol-naïve GABA_B(1)_ WT and KO mice. Unpaired *t*-test (*p < 0.05) (*n* = 3/group). All data are expressed as mean ± SEM.

Because of the important changes in GABA_A_R gene expression found in the GABA_B(1)_ KO mice, we studied the GABAergic inhibitory components in the hippocampus. To this end, we targeted DGGCs, a neuronal cell population characterized by a prominent tonic current that is primarily mediated by extrasynaptic δ-GABA_A_R. DGGCs also display a phasic inhibitory component attributable to the activation of ɣ2-containing GABA_A_R that are located at synaptic level (Nusser and Mody, 2002; Stell and Mody, 2002). Under our experimental conditions, the activation of GABA_A_R caused by spontaneous release of GABA from presynaptic terminals caused a consistent inward current reflecting an outflow of Cl- ions. After a recording of 3 min for a stable baseline, the hippocampal slices were perfused for 6 min with THIP, resulting in consistent increase in noise variance and negative shift of the holding current with respect to the baseline. Interestingly, despite the decrease in δ subunit abundance observed in the GABA_B(1)_ KO mice, we found no difference in increase in tonic current with THIP compared to the WT animals as revealed by a similar holding current shift (unpaired *t*-test *t* = 0.1848, df = 13; *p* = 0.8562), and change in noise variance (unpaired *t*-test *t* = 1.623, df = 13; *p* = 0.1286) (Figures 4A–C); in agreement, the subsequent bicuculline application to the bath ended all GABAergic currents causing the expected positive shift of baseline as well as reduction in noise variance in a similar extent in both the WT and KO mice (Figures 4A–C). On the contrary, analyses of sIPSC kinetics revealed a significant increase in the amplitude of phasic inhibitory transmission (unpaired *t*-test, *t* = 2.645, df = 12; *p* = 0.0214) (Figures 4D,E). Conversely, no changes in sIPSC frequency (unpaired *t*-test, *t* = 1.086, df = 12; *p* = 0.2987) and decay time (unpaired *t*-test, *t* = 0.5393, df = 12; *p* = 0.5995) were noted. Supporting the increase in the amplitude of phasic inhibitory current, we found a significant increase in ɣ^2^ subunit abundance in the hippocampus of the GABA_B(1)_ KO mice compared with the WT littermates (unpaired *t*-test, *t* = 5.018, df = 6; *p* = 0.0024) (Figure 4F) that provides a rationale for the increase in the amplitude of the phasic inhibitory component.

### Voluntary Alcohol Consumption Differently Affects GABA_A_R Subunits Gene Expression in the Hippocampus of GABA_B(1)_ KO and HZ Mice

**FIGURE 5:**
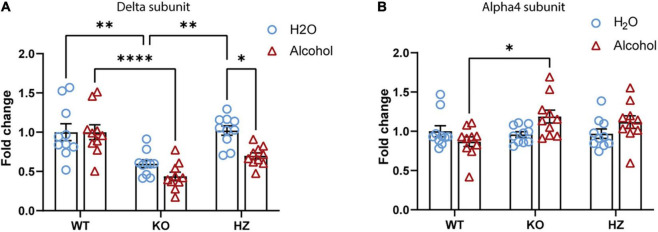
Effect of voluntary alcohol consumption on GABA_A_ receptor gene expression. **(A)** Delta subunit mRNA level in the hippocampus of GABA_B(1)_ WT, KO, and HZ and mice at the end of the last drinking session of 2BC. **(B)** Bar graphs represent the analysis of alpha4 subunit mRNA level in the hippocampus of GABA_B(1)_ WT, KO, and HZ mice at the end of the last drinking session of 2BC. All data are expressed as mean ± SEM; (*n* = 10/group). *Post hoc* test (*p < 0.05; ^**^p < 0.01; ^****^p < 0.0001).

We have previously documented that voluntary alcohol consumption is associated with increase in the abundance of hippocampal GABA_A_R δ subunit at the mRNA and protein levels (Sanna et al., 2011; Follesa et al., 2015). In this study, we investigated whether voluntary alcohol consumption can revert the dramatic downregulation of hippocampal GABA_A_R δ subunit found in alcohol naïve-GABA_B(1)_ KO mice. Thus, we measured after the last drinking session the expression level of GABA_A_R δ and its associated partner subunit α4 in the hippocampus of alcohol-exposed GABA_B(1)_ KO, HZ, and WT mice and relative controls (water group). Surprisingly, we found that exposure to the 2BC drinking paradigm did not revert the dramatic downregulation of the hippocampal δ subunit in the GABA_B1_ KO mice but rather exacerbated this condition. Accordingly, the HZ and GABA_B(1)_ mice exposed to voluntary alcohol drinking showed the same condition of KO mice with robust reduction in the δ subunit. Conversely, the alcohol-exposed WT mice did not show any change in GABA_A_R δ subunit expression. The two-way ANOVA revealed a main effect of genotype [*F*_(2,53)_ = 25.32, *p* < 0.0001] and treatment [*F*_(1,53)_ = 13, *p* = 0.0007]; the *post hoc* test revealed a significant decrease in δ subunit abundance in the HZ alcohol-exposed mice compared to the water-exposed controls (Tukey’s multiple comparisons test; *p* = 0.0084) (Figure 5A). Intriguingly, we found an overall increase in GABA_A_R α4 subunits in GABA_B1_ KO and HZ compared to the WT mice, which, on the contrary, presented a slight decrease in this subunit. The two-way ANOVA showed a significant interaction (genotype × time) [*F*_(2,54)_ = 4.001, *p* = 0.024], but no main effects were found. Tukey’s multiple comparisons test revealed a significant increase in α4 subunits in the alcohol-exposed GABA_B(1)_ KO mice compared to the alcohol-exposed WT controls (*p* = 0.0163) (Figure 5B).

### Lack of Steroidogenic Activity Following Alcohol Administration in GABA_B(1)_ KO Mice

**FIGURE 6:**
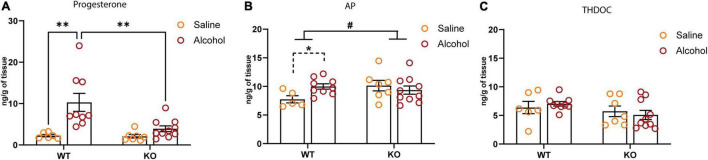
Effect of systemic alcohol administration on brain level of progesterone, 3α,5α-TH-PROG (allopregnanolone, AP), and 3α,5α-tetrahydrodeoxy corticosterone (THDOC). **(A)** Levels of progesterone in the hippocampus of WT and KO mice 60 min after systemic alcohol or saline administration. *Post hoc* test (^**^p < 0.01) (WT saline, *n* = 5; KO saline, *n* = 6; WT alcohol, *n* = 9; KO alcohol, *n* = 10). **(B)** Levels of AP in the hippocampus of WT and KO mice 60 min after systemic alcohol or saline administration. Genotype × treatment interaction is indicated with solid lines (#*p* < 0.05); unpaired *t*-test is indicated with dashed lines (*p < 0.05) (WT saline, *n* = 5; KO saline, *n* = 7; WT alcohol, *n* = 9; KO alcohol, *n* = 10). **(C)** Levels of THDOC in the hippocampus of WT and KO mice 60 min after systemic alcohol or saline administration (WT saline, *n* = 6; KO saline, *n* = 7; WT alcohol, *n* = 9; KO alcohol, *n* = 10). All data are expressed as mean ± SEM.

Because of the potential involvement of GABA_B_R in regulating the levels of neuroactive steroids and the established role of these compounds in the regulation of δ-GABA_A_R and alcohol drinking (Sundstrom-Poromaa et al., 2002; Stell et al., 2003; Wallner et al., 2003; Morrow et al., 2006; Jensen et al., 2017), we profiled the hippocampal neuroactive steroid levels in the GABA_B(1)_KO and WT mice following alcohol administration. To this end, we measured the levels of GABAergic neurosteroids 3α-hydroxy-5α-pregnan-20-one (3α,5α-TH-PROG; allopregnanolone, AP), 3α,5α-tetrahydrodeoxycorticosterone (3α,5α-THDOC; THDOC), and progesterone. Interestingly, we did not report any significant change in the basal level of the neuroactive steroids profiled. On the other hand, we found that the single dose (1.5 mg/kg) of alcohol significantly increased the hippocampal level of progesterone in WT but not KO; the two-way ANOVA analyses showed a main effect of treatment [*F*_(1,28)_ = 13.22, *p* = 0.0011] and genotype [*F*_(1,28)_ = 5.788, *p* = 0.023] as well as interaction between the two factors [*F*_(1,28)_ = 5.24, *P* = 0.0298] (Figure 6A). We also found a significant genotype × treatment interaction [*F*_(1,27)_ = 4.276, *p* = 0.0484] for the neuroactive steroid AP, reflecting a different response of GABA_B(1)_ KO to alcohol administration; however, no significant main effects of either genotype or treatment were detected. To better evaluate the effects of alcohol on neuroactive steroid AP, in WT alcohol-treated compared with WT saline controls time (unpaired *t*-test *t* = 2.861, df = 12; *p* = 0.0143), but not in the KO alcohol-exposed mice compared with the KO saline controls (unpaired *t*-test *t* = 0.6913, df = 15; *p* = 0.4999) (Figure 6B). On the contrary, the two-way ANOVA did not reveal any effects of acute alcohol administration on the hippocampal level of THDOC (Figure 6C).

## Discussion

The metabotropic ɣ-aminobutyric acid type B receptor (GABA_B_R) system represents one of the most promising targets for effective treatment of AUDs. GABA_B_R agonists such as baclofen and GHB have been often proposed as anti-alcohol therapy (Caputo et al., 2009; Agabio et al., 2018). However, their use in clinics has been limited because of the presence of side effects including sedation, cognitive disruption, and, in the case of GHB, abuse liability and possibility of accidental overdose (Carter et al., 2006; Augier, 2021). An alternative approach to GABA_B_R agonists may be represented by positive allosteric modulators of the GABA_B_R that have shown similar therapeutic efficacy compared to agonists and better side-effect profile (Filip et al., 2015). However, clinical validation of these compounds is warranted. More broadly, the possibility to disrupt the acute side effect of GABA_B_R modulators without abolishing the positive effect on reducing alcohol drinking will have an implication for treatment in clinics. To this end, a better understanding of GABA_B_R-associated pathways involved in alcohol drinking is required to design more selective and effective therapeutic strategies. Nonetheless, only few investigations on mechanisms by which this receptor regulates alcohol drinking have been accomplished thus far. In this study, we investigated for the first-time the alcohol drinking behavior of mice lacking functional GABA_B_R with the 2-bottle choice (2BC) paradigm, and then we analyzed the expression and function of extrasynaptic δ-GABA_A_Rs that are functionally related to GABA_B_Rs in brain areas relevant to alcohol drinking and dependence (Jia et al., 2008; Enoch et al., 2012; Diaz et al., 2014; Ma et al., 2022).

The 2BC paradigm represents a useful tool to study the willingness of an experimental animal to consume alcohol and allows for determination of a preference for an alcoholic solution over water (Crabbe et al., 2010). We found that the GABA_B(1)_ KO and HZ mice drank higher amounts of the alcohol solution, preferred alcohol to water, and had higher BACs compared to their WT littermates. To date, the total amount of alcohol consumed by GABA_B(1)_ KO and HZ mice is substantially lower than what our group and others have found using the C57 mouse strain that, under similar conditions, showed higher alcohol intake and BAC (Griffin et al., 2007, 2009; Follesa et al., 2015). However, it has been reported that different mouse strains show a variety of intakes with 2BC, and that the C57 strain appears to be the one with highest alcohol intake. It has been shown that the C57 mouse strain consumes roughly five times more alcohol compared to mice having the same genetic background as the subjects used in our study (Yoneyama et al., 2008; Rosenwasser et al., 2013). Thus, the natural proclivity of certain mouse strains to consume larger amounts of alcohol may account for this difference.

Since previous studies have shown that the GABA_B_R agonist GHB is effective in diminishing voluntary alcohol drinking in animal models and human patients with AUD (Biggio et al., 1992; Gallimberti et al., 1992), and loss of pharmacological effects of GHB have been previously reported in GABA_B(1)_ KO mice (Kaupmann et al., 2003), we evaluated the effect of GHB on 2BC test in our experimental groups. As expected, we found that GHB did not change alcohol intake in the GABA_B(1)_ KO mice but significantly reduced the alcohol consumption in the HZ and WT mice. Interestingly, we found that the effect of GHB on reducing alcohol drinking was less in the WT mice than in the HZ mice. However, this agrees with previous studies showing that the anti-alcohol effect of GABA_B_R agonists (baclofen and GHB) are more effective in animals consuming higher levels of alcohol or in alcohol-experienced animals (Colombo et al., 2002, 2012; Agabio and Colombo, 2014). We also ruled out the possibility that the reduction in alcohol intake was due to secondary effects (i.e., sedation), because a corresponding dose of GHB did not reduce spontaneous locomotor activity in an independent cohort of mice.

An investigation of the role of GABA_B_R in regulating alcohol drinking revealed molecular and functional changes in GABA_A_Rs in the hippocampus of the GABA_B(1)_ KO mice. The hippocampus is considered an important component of brain reward pathways, a complex brain circuit associated with development of drug addiction (Morón et al., 2007; Koob and Volkow, 2010; Werner et al., 2020). In particular, it is important for the association between the drug context and the rewarding properties of drug of abuse facilitating the transition from drug use to dependence (Kutlu and Gould, 2016). The involvement of the hippocampal GABAergic system in alcoholism has been confirmed with compelling evidence by several studies (Cagetti et al., 2003; Liang et al., 2006, 2007; Shen et al., 2011; Enoch et al., 2012; Follesa et al., 2015). An RNA-seq analysis revealed a reduction of GABA_B(1)_ in the hippocampus of human alcoholics and cocaine addicts and alcohol-naïve alcohol-preferring rats that was concurrent with plastic changes in GABA_A_R (Enoch et al., 2012). Interestingly, the increase in ɣ^2^ subunit abundance that we found in our study confirmed what has been found in the hippocampus of alcohol-preferring rats and human alcoholics and cocaine addicts in the opposite direction (Enoch et al., 2012), suggesting that homeostatic changes in hippocampal GABA_A_R associated with GABA_B_R loss have common features across different species, genetic backgrounds, and drug usage conditions. However, the analysis by Enoch et al. (2012) failed to detect the expression level of the δ subunit that appears to be expressed at low levels and segregated in discrete areas of the hippocampus such as the dentate gyrus (Wei et al., 2004). For these reasons, it may not have been detected by a bulk RNA-seq analysis that considered the whole hippocampus and heterogeneous cell population. Nonetheless, loss of δ GABA_A_R subunit in the hippocampus has been documented by previous studies on chronic intermittent ethanol (CIE) exposed rats and during alcohol withdrawal (Cagetti et al., 2003; Liang et al., 2006), suggesting that loss of this subunit is associated with excessive alcohol-drinking. In contrast, whole-body δ-deficient mice displayed reduced alcohol consumption and preference in a 24-h access 2BC paradigm (Mihalek et al., 2001). It has been suggested that regional differences in which loss GABA_A_R subunit δ occur may account for this discrepancy. Site-specific pharmacological manipulation and loss of function studies appear to be consistent with this hypothesis (Nie et al., 2011; Fritz and Boehm, 2014; Melón et al., 2019). Site-specific loss of function studies targeting the hippocampus may help to address this important point.

To understand how these molecular changes may affect cellular function, we recorded GABAergic inhibitory components in the DGGCs of alcohol naïve GABA_B(1)_ KO and WT mice. Surprisingly, the downregulation of the δ subunit did not result in decrease in inhibitory tonic currents in DGGCs that, on the contrary, were maintained in the mice. However, our analysis on tonic currents agreed with a previous study that, with the same mouse model and analogous experimental conditions, has found superimposable outcomes (Connelly et al., 2013). On the other hand, we found an increase in the amplitude of spontaneous inhibitory postsynaptic currents (sIPSCs) in DGGCs of the GABA_B(1)_ KO mice, providing a functional correlation with the increased abundance of the ɣ^2^ subunit. Thus, we hypothesized that loss of GABA_B_R function, which has been predicted to result in elevated levels of extracellular GABA (Waldmeier et al., 2008; Enoch et al., 2012), may lead to reduction in the expression of δ-containing GABA_A_Rs that are sensitive to low extracellular GABA, and subsequent replacement of these receptors with ɣ^2^-containing GABA_A_R. Therefore, these homeostatic modifications may account for the balance between phasic (synaptic) and tonic (extrasynaptic) inhibition shifting toward the direction of phasic inhibition. Consistently, it has been documented that elevated extracellular GABA levels may result in tonic activation of synaptic ɣ^2^-containg GABA_A_R, providing a plausible mechanism by which tonic currents are maintained in the face of low extrasynaptic δ-GABA_A_R availability but high ambient GABA levels (Jensen et al., 2003; Mortensen and Smart, 2006; Zhang et al., 2007; Rajasekaran et al., 2010). Alternatively, since mRNA expression abundance does not always correlate with protein levels, it is possible that δ protein amount does not suffer a dramatic downregulation; therefore, the number of receptors containing this subunit may remain unchanged. For this reason, we cannot rule out that δ-containing GABA_A_Rs are sustaining tonic currents. It is worth to mention that we have previously documented that protein levels for the δ subunit appear to be in the same direction of the mRNAs in C57 social-isolated mice (Sanna et al., 2011). Moreover, it has been found that hetero-oligomerization between GABA_B_R and GABA_A_R regulates receptor trafficking and crosstalk (Balasubramanian et al., 2004). Therefore, lack of GABA_B_R may affect GABA_A_R exposition in the membrane, irrespective of subunit availability and abundance.

**FIGURE 7:**
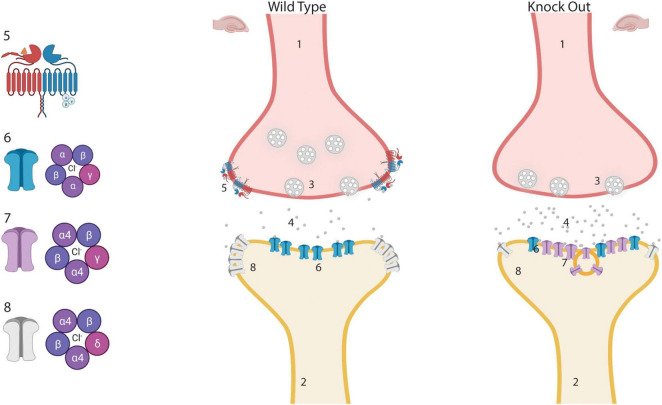
Potential modification of inhibitory synapses in the hippocampus of GABA_B(1)_ knock out (KO) mice. Lack of GABA_B_ autoreceptor may produce an increase in the release of GABA from the presynaptic terminal. Chronically increased GABA may generate a downregulation of extrasynaptic α4βδ-GABA_A_ receptors and upregulation of newly formed synaptic α4βγ-GABA_A_ receptors that are less sensitive to GABA but sensitive to low alcohol concentrations. We hypothesized that the presence of α4β ɣ^2^-GABA_A_Rs in the synaptic compartment may increase the preference for consuming alcoholic solutions. (1) Presynaptic terminal. (2) Postsynaptic terminal. (3) Synaptic vesicle. (4) Extracellular GABA. (5) GABA_B_ receptor. (6) αβγ-GABA_A_ synaptic receptors. (7) Newly formed synaptic α4β ɣ^2^-GABA_A_ receptor. (8) Extrasynaptic α4βδ-GABA_A_ receptors. This Figure was created with “BioRender.com” (OJ23XQT9O0).

Reasoning on how these molecular and functional changes in the hippocampus of the GABA_B(1)_ KO mice may impact alcohol consumption and dependence, we found that imbalance in the expression of hippocampal δ (downregulated) and ɣ^2^ (upregulated) subunits has been described after different alcohol intoxication paradigms *in vivo* and *in vitro*, and in different animal models of chronic epilepsy (Liang et al., 2006, 2007; Lagrange et al., 2007; Sun et al., 2007; Rajasekaran et al., 2010; Shen et al., 2011; Grabenstatter et al., 2014). In native GABA_A_R, the γ2 and δ subunits seems to be mutually exclusive; therefore, they may compete for partnership with the α4 subunit (Shivers et al., 1989; Araujo et al., 1998; Sur et al., 1999; Martenson et al., 2017). Thus, the condition of decreased δ and elevated γ2 availability could favor the partnership of the latter with the α4 subunit, giving origin to a new α4/ ɣ^2^-containing GABA_A_R (α4/ ɣ^2^-GABA_A_R) with different physiological and pharmacological responses. It has been shown that the presence of hippocampal α4/ ɣ^2^-GABA_A_Rs in the synaptic compartment may potentiate synaptic but not tonic inhibition in animal models of chronic epilepsy and after CIE exposure in rats; therefore, their presence has been associated with development of alcohol dependence and with seizure susceptibility (Cagetti et al., 2003; Cohen et al., 2003; Peng et al., 2004; Liang et al., 2006, 2007; Sun et al., 2007). Several other characteristics of α4/ ɣ^2^-GABA_A_R have been described in recent studies. It has been found that this receptor appears to be less sensitive to extracellular GABA (Zhang et al., 2007; Rajasekaran et al., 2010), providing a functional explanation to the occurrence of this homeostatic response in conditions associated with elevated ambient GABA level such as lack of presynaptic GABA_B_ autoreceptors (Waldmeier et al., 2008) or during excessive GABA_A_R stimulation following alcohol exposure (Davies, 2003). α4/ ɣ^2^-GABA_A_R also showed less responsiveness to the modulatory action of benzodiazepine and neurosteroids (Cohen et al., 2003; Liang et al., 2007; Rajasekaran et al., 2010; Shen et al., 2011), two potent anxiolytic compounds capable of changing the behavioral response of alcohol consumption (Mueller et al., 2005; Morrow et al., 2006). Conversely, α4/ ɣ^2^-GABA_A_R appears to be more sensitive to low alcohol concentration and its anxiolytic effect (Liang et al., 2006). For this reason, this receptor was proposed to represent the physiological substrate of persistent alcohol-induced anxiolytic effects, a condition that, in CIE rats and human alcoholics, leads to increased preference to alcohol consumption (Liang et al., 2006). In accordance with these findings, the GABA_B(1)_ KO mice showed augmented anxiety and reduced response to the behavioral effect of diazepam (Mombereau et al., 2004), spontaneous epileptiform activity (Schuler et al., 2001), increased synaptic inhibition, and increased voluntary alcohol consumption and preference (current study). We summarize the potential modification of the GABAergic synapse that may take place in the hippocampus of the GABA_B(1)_ KO mice in Figure 7.

Different patterns of alcohol exposure in animal models it has been shown to produces plasticity in the GABAergic system *via* regulation of gene expression (Olsen and Liang, 2017). A previous study from our group revealed that exposure to the 2BC paradigm resulted in increase in the abundance of the hippocampal δ subunit (Sanna et al., 2011; Follesa et al., 2015). Therefore, we investigated whether voluntary alcohol consumption can revert the loss of this subunit found in alcohol-naïve GABA_B(1)_ KO mice. Surprisingly, voluntary alcohol consumption did not reverse the dramatic loss of the δ subunit but rather exacerbates this condition in the GABA_B(1)_ KO mice. Interestingly, also, the HZ mice exposed to voluntary alcohol drinking suffered a robust downregulation of the δ subunit, most likely precipitating in the condition we described for the GABA_B(1)_ KO mice, with gain of synaptic GABA_A_R responsiveness to alcohol that is considered the hallmark of long-term alcohol dependence (Enoch, 2008; Olsen and Liang, 2017). Consistently with this idea, we found that voluntary alcohol consumption is associated with overall increase in the α4 subunit in the GABA_B(1)_ KO and HZ mice, a homeostatic change that is associated with long-term dependence in alcoholics (Jin et al., 2012) and increased anxiety in CIE and socially isolated rats (Cagetti et al., 2003; Serra et al., 2006). Thus, upregulation of α4 and downregulation of the δ subunit support the likelihood of increased α4/ ɣ^2^-GABA_A_R formation, suggesting a conceivable mechanism by which alcohol dependence may be sustained in the GABA_B(1)_ KO and HZ mice. Another potential mechanism by which GABA_B_R could affect both GABA_A_R transmission and alcohol consumption has been suggested by a previous study showing a possible involvement of GABA_B_R in mechanisms that mediate increase in brain level of neuroactive steroid following GHB administration (Barbaccia et al., 2002). Neuroactive steroids are an important class of endogenous modulators that potently affect GABAergic neurotransmission preferentially through extrasynaptic δ-GABA_A_R (Biggio et al., 2007). Studies on rodents have documented alcohol-induced neuroactive steroids significantly contribute to the behavioral effects of alcohol such as anxiolytic (Hirani et al., 2005), antidepressant (Hirani et al., 2002), and sedative–hypnotic (VanDoren et al., 2000; Khisti et al., 2003), raising the possibility that lack of steroidogenic response may favor increased alcohol drinking behaviors. Remarkably, we found that alcohol administration produced steroidogenic activity in WT but not in the GABA_B(1)_ KO mice, suggesting that the presence of GABA_B_R is instrumental to increase neuroactive steroid levels following alcohol administration. Specifically, we found a robust increase of progesterone in the hippocampus of WT but not the GABA_B(1)_ KO mice following alcohol administration and a milder but still significant effect on the neurosteroid AP. On the contrary, irrespective of genotype, no effects of alcohol administration on neurosteroid THDOC were noted. Our results on the WT mice agree with previous findings showing that acute ethanol administration elevates progesterone level in mice serum (Porcu et al., 2010). In contrast, we found different effects on AP, which has been found to be reduced in the serum of C57BL/6J or unchanged in DBA/2J mice under similar experimental conditions (Porcu et al., 2010). However, other studies have reported increased brain level of AP following alcohol administration (Gabriel et al., 2004), suggesting that the serum and brain levels of neuroactive steroids may be regulated differently. Pointing in this direction, it has been found that the activity of the 5a-reductase enzyme family, required for conversion of progesterone into 5α-dihydroprogesterone (a key step in the production of neuroactive GABAergic steroids), appears to be sensitive to regional differences with intense level of activity in the mouse hippocampus (Roselli and Snipes, 1984). However, irrespective of these differences, it is important to emphasize that the lack of alcohol-induced steroidogenesis in the GABA_B(1)_ KO mice may be an important factor for maintaining preference for alcohol consumption. Given the importance of steroidogenesis pathways in mediating several behavioral consequences of alcohol consumption, it is conceivable that lack of alcohol-induced steroidogenesis could be a potent driver to consume greater amount of alcohol that may be necessary to produce relevant pharmacological effects that, in the presence of steroidogenic activity, are achieved with lower alcohol levels. Supporting this hypothesis, it has been reported that diminished steroidogenic activity was found in alcohol-tolerant and alcohol-dependent animal models (Morrow et al., 2001). It is worthwhile to mention that the basal level of neurosteroids was not affected by genotype, suggesting that facilitation of the steroidogenic activity mediated by GABA_B_R only takes place under specific conditions such as GHB administration (Barbaccia et al., 2002) or following alcohol administration (current study). Future studies will be aimed to investigate the role of GABA_B_R in stress-mediated steroidogenesis that may be of importance for severe clinical conditions such as depression and post-traumatic stress disorder (Cryan and Kaupmann, 2005; Pinna, 2020).

In summary, the findings of this study documented that lack of GABA_B(1)_ results in increased alcohol consumption and preference, corroborating previous preclinical and clinical studies on the role of this receptor in modulation of alcohol drinking behaviors and dependence (Biggio et al., 1992; Colombo et al., 2002; Addolorato et al., 2011). To the best of our knowledge, this is the first study that investigates GABA_A_R subunit gene expression and functions in GABA_B(1)_ KO mice, providing new evidence that complements previous behavioral, molecular, and electrophysiological data on GABA_B_/GABA_A_ interactions at the functional level (Mombereau et al., 2004; Enoch et al., 2012; Connelly et al., 2013). The plastic and functional changes associated with GABA_A_R that occurred in the hippocampus of the GABA_B(1)_ KO mice support previous findings on the hippocampus of rats exposed to the CIE model or after acute alcohol intoxication (binge model) with net loss of extrasynaptic and gain of aberrant synaptic GABA_A_R that is considered the hallmark of long-term alcohol dependence (Cagetti et al., 2003; Liang et al., 2006, 2007). Finally, our study revealed a previously overlooked role of GABA_B_R in regulation of alcohol-induced neuroactive steroids that may play an important role in mediating some behavioral effects produced by alcohol; therefore, lack of steroidogenic activity may increase preference for alcohol consumption (Morrow et al., 2006). Nonetheless, caution should be taken while interpreting the data, and we acknowledge potential limitations. First, we do not have direct evidence showing that loss of GABA_B_R in the hippocampus is specifically responsible for maintaining alcohol drinking behaviors, and we cannot exclude that this effect may be produced and/or contributed by lack of this receptor in other brain regions. Functional studies including specific loss of function approaches are required to address this point and to confirm our data as well as previous findings on human alcoholics and alcohol-preferring rats (Enoch et al., 2012). Second, our molecular analysis was limited to mRNAs, and we did not evaluate protein levels and the effective composition of GABA_A_Rs, making difficult to draw final conclusions on how lack of GABA_B_R impacts GABA_A_R configuration and function. Moreover, our data on the ɣ^2^ subunit was limited to naïve animals, and we did not extend the analysis to alcohol-exposed animals. Nonetheless, our molecular data were supported by the electrophysiological recording pointing to a shift in the direction of phasic inhibition according to previous studies showing similar results in animal models of excessive alcohol consumption and chronic epilepsy (Sur et al., 1999; Cagetti et al., 2003; Cohen et al., 2003; Liang et al., 2006, 2007). Future studies should be devoted to analyzing the potential differences in functional responses to different alcohol concentration, in the presence of these homeostatic modifications. Finally, we studied the role of GABA_B_R in alcohol-induced neuroactive steroids only in the context of acute alcohol challenge, and we did not evaluate the impact of GABA_B_R loss on neuroactive steroid level during chronic alcohol consumption. Since previous studies have shown that alcohol consumption and alcohol injection produced different outcomes on brain levels of GABAergic steroids (Finn et al., 2004), future studies should consider profiling neuroactive steroids following acute and/or chronic alcohol drinking to better understand how steroidogenic pathways affect voluntary alcohol consumption in GABA_B(1)_ KO mice. Nonetheless, irrespective of these potential limitations, our study underscores the role of GABA_B_R in alcohol drinking behaviors, highlighting the molecular and functional changes occurring in the hippocampus of GABA_B(1)_ KO mice and offering a plausible mechanism by which preference for alcohol consumption is maintained in these mice. Moreover, the lack of alcohol-induced steroidogenic activity in the GABA_B(1)_ KO mice suggests that functional interactions among GABA_B_R, neuroactive steroids, and GABA_A_R may be more complex and intertwined than previously appreciated.

## Data Availability Statement

The original contributions presented in the study are included in the article/supplementary materials, further inquiries can be directed to the corresponding authors.

## Ethics Statement

The animal study was reviewed and approved by the “Committee of Animal Use and Care” of University of Cagliari.

## Author Contributions

GF and PF designed the experiments and wrote the manuscript. GF, GA, GT, FB, MP, LC, MZ, and EM performed the experiments, monitored data collection, and performed statistical analysis. MS contributed to the design of the experiments, monitored data collection, and supervised the experiment execution with PF. All authors contributed to data interpretation, revised the manuscript, and approved the final version.

## Conflict of Interest

The authors declare that the research was conducted in the absence of any commercial or financial relationships that could be construed as a potential conflict of interest.

## Publisher’s Note

All claims expressed in this article are solely those of the authors and do not necessarily represent those of their affiliated organizations, or those of the publisher, the editors and the reviewers. Any product that may be evaluated in this article, or claim that may be made by its manufacturer, is not guaranteed or endorsed by the publisher.
